# Clinical Effects of RUNX1 Mutations on the Outcomes of Patients with Acute Myeloid Leukemia Treated with Allogeneic Hematopoietic Stem-Cell Transplantation

**DOI:** 10.3390/curroncol32060294

**Published:** 2025-05-22

**Authors:** Wei-Jie Ran, Lan-Ping Xu, Xiao-Hui Zhang, Ying-Jun Chang, Xiao-Dong Mo, Yu-Qian Sun, Xiao-Jun Huang, Yu Wang

**Affiliations:** Peking University People’s Hospital, Peking University Institute of Hematology, National Clinical Research Center for Hematologic Disease, Beijing Key Laboratory of Hematopoietic Stem Cell Transplantation, Collaborative Innovation Center of Hematology, Peking University, Beijing 100044, China; rwjpku123@sina.com (W.-J.R.); lpxu_0415@sina.com (L.-P.X.); zhangxh100@sina.com (X.-H.Z.); rmcyj@bjmu.edu.cn (Y.-J.C.); mxd453@163.com (X.-D.M.); sunyuqian83@hotmail.com (Y.-Q.S.); xjhrm@medmail.com.cn (X.-J.H.)

**Keywords:** acute myeloid leukemia, hematological malignancies, haploidentical hematopoietic stem cell transplantation, RUNX1 gene

## Abstract

It is reported that AML with RUNX1 mutations is associated with poorer response to conventional chemotherapy, lower rates of complete remission (CR), leukemia-free survival (LFS), and overall survival (OS). We aimed to evaluate the prognostic impact of RUNX1 mutations following allogeneic hematopoietic stem cell transplantation (allo-HSCT) by comparing clinical outcomes in AML patients with and without RUNX1 mutations. We retrospectively analyzed 91 AML patients (33 RUNX1+ and 58 RUNX1−) who received their first HSCT at Peking University People’s Hospital. The median age of the cohort was 38 years (range: 6–64), with 73 patients (80%) receiving Haploidentical HSCT and 18 patients (20%) receiving sibling-matched allo-HSCT. In univariate analyses, no significant differences in survival outcomes were observed. For the RUNX1-mutation group and RUNX1-wild-type group, the 2-year cumulative incidence of relapse (CIR) was (12.6% vs. 7.6%, *p* = 0.472), the 2-year non-relapse mortality (NRM) rate was (9.6% vs. 7.2%, *p* = 0.747), the 2-year LFS was (77.8% vs. 85.2%, *p* = 0.426), and the 2-year OS rate was (85.9% vs. 92.7%, *p* = 0.397). We did not observe any negative impact of RUNX1 mutations on clinical outcomes, suggesting that allo-HSCT (especially Haplo-HSCT) may mitigate the adverse prognostic influence of RUNX1 mutations in AML.

## 1. Introduction

The *RUNX1* gene, located on chromosomal band 21q22, plays a critical role in the formation of the hematopoietic system, and mutations in this gene are associated with various hematological malignancies, including acute myeloid leukemia (AML) [1,2]. *RUNX1*-mutated AML presents a distinct cytogenetic profile, often featuring trisomy 8 or 13 and additional molecular mutations such as *ASXL1* and *SRSF2* [3,4,5,6]. These cases typically lack *NPM1* and CEBPA double mutations, highlighting their unique genetic characteristics. *RUNX1* mutations have been confirmed as vital prognostic markers in AML, informing risk stratification in numerous studies. In the 2022 ELN guidelines, *RUNX1* mutations were classified as indicative of poor prognosis [7]. Due to its distinct clinical features, the 2016 WHO classification recognized AML with *RUNX1* mutations as a provisional entity [3,5,6,8,9]. However, in the 2022 WHO classification, *RUNX1*-mutated AML was removed as a standalone entity due to its overlap with a broad range of other molecular characteristics, making it insufficiently specific to define a unique subtype of AML [10].

*RUNX1* mutations are consistently associated with a poor prognosis, contributing to lower rates of complete remission (CR), leukemia-free survival (LFS), and overall survival (OS) compared to wild-type *RUNX1* AML [3,6,8,11,12,13]. Allogeneic hematopoietic stem cell transplantation (allo-HSCT) remains a critical curative approach for high-risk AML patients. Studies by Chan et al. [14] and Della Porta et al. [15] suggest that AML patients with RUNX1 mutations experience reduced OS and increased relapse rates following allo-HSCT. In contrast, Waidhauser et al. [16] reported no significant effect of RUNX1 mutations on post-transplant outcomes in AML patients. Conversely, Chou et al. [17] observed a favorable prognostic impact of RUNX1 mutations in AML patients undergoing allo-HSCT, while Gaidzik et al. [13] found improved relapse-free survival (RFS) in RUNX1+ AML patients post-transplantation. The existing data on the prognostic influence of RUNX1 mutations in the context of allo-HSCT remain limited, inconsistent, and frequently conflicting, largely derived from small subgroup analyses. Consequently, further research is essential to elucidate the impact of RUNX1 mutations on post-transplant outcomes in AML patients.

Haploidentical HSCT (Haplo-HSCT) has emerged as a viable alternative for patients without an HLA-matched donor or those requiring urgent transplantation [18,19]. Current evidence suggests that Haplo-HSCT offers therapeutic outcomes comparable to HLA-matched sibling donor transplantation, unrelated donor transplantation, and umbilical cord blood transplantation [20,21]. Also, for high-risk populations, especially for pre-HSCT MRD-positive patients, Haplo-HSCT can mediate a more substantial graft-versus-leukemia (GVL) effect than HLA-matched sibling donor transplantation [22,23,24], making it a promising treatment option for AML patients with *RUNX1* mutations. However, the previous studies did not specifically focus on the subgroup of AML patients with *RUNX1* mutations who underwent Haplo-HSCT. This raised our interest in exploring the potential of Haplo-HSCT to improve the poor prognosis typically associated with *RUNX1*-mutated AML. This study aims to evaluate the clinical outcomes of AML patients with *RUNX1* mutations who received allo-HSCT (especially Haplo-HSCT).

## 2. Materials and Methods

### 2.1. Patients

This retrospective study was endorsed by the ethics committee of Peking University People′s Hospital and was granted a waiver for informed consent. In this study, we selected patients with AML who underwent their first allo-HSCT at the Peking University Institute of Hematology between January 2020 and December 2021, with complete information on RUNX1 mutation status. Complete RUNX1 mutational data were available at diagnosis for all patients. Patients who had received a second transplant or exhibited the t(8;21)/RUNX1-RUNX1T1 translocation were excluded.

### 2.2. Detection of RUNX1 Mutations

All enrolled patients were screened for mutations of *RUNX1* at the time of diagnosis and received next-generation sequencing (NGS). The testing methods have been briefly described earlier [25,26].

### 2.3. Transplant Protocols

The conditioning regimen and graft-versus-host disease (GVHD) prophylaxis were implemented in accordance with the methodologies previously described by our center [27]. All patients received a Busulfan+ Cyclophosphamide (BuCy)-based conditioning regimen. The conditioning regimen contained cytarabine (2 g/m^2^/day IV, days −10 and −9), busulfan (3.2 mg/kg/day, intravenously, days −8 to −6), cyclophosphamide (1.8 g/m^2^/day, days −5 to −4), semustine (250 mg/m^2^, day −3), and rabbit ATG (thymoglobulin; Imtix Sangstat, Lyon, France; 2.5 mg/kg/day, days −5 to −2). Haplo-HSCT and unrelated patients received ATG. Patients who received an HLA-matched sibling donor transplantation were treated with a regimen identical to that of the patients who received an HLA-Haploidentical HSCT, except that ATG was not required. Cyclosporine A, mycophenolate mofetil, and short-term methotrexate were applied for GVHD prophylaxis.

### 2.4. Definitions and End-Points

OS was calculated from the date of AML diagnosis to the date of death. Non-relapse mortality (NRM) was defined as the interval from diagnosis to death in the absence of relapse, while LFS was determined as the period from diagnosis to either relapse or death without prior relapse. Relapse was characterized as the recurrence of hematological disease [28]. Complex karyotype (CK) was identified as the presence of three or more chromosomal abnormalities [28]. The HCT-CI score, aGVHD, and cGVHD were categorized according to established criteria [29,30,31]. The composite endpoint of GVHD-free, relapse-free survival (GRFS) was defined as survival post-transplantation without Grade III–IV aGVHD, cGVHD requiring treatment, or relapse [32]. MRD was evaluated using multiparameter flow cytometry with a standardized clinical panel 3 to 4 weeks before HSCT [33,34].

### 2.5. Statistical Analysis

The two cohorts’ patient, disease, and transplant-related characteristics (*RUNX1+* and *RUNX1−*) were compared using the χ^2^ or Fisher’s exact test for categorical variables and the Mann–Whitney U-test for continuous variables. Continuous variables were categorized for univariate analysis, and the median value was used as a cut point; the log-rank and Fine–Gray’s tests were used to compare outcomes between the groups [35]. All tests were two-sided. The SPSS24.0 (IBM Corp, Armonk, NY, USA) and R4.2.2 (https://www.Rproject.org (accessed on 23 August 2023)) were used to perform the analyses and plots. 

## 3. Results

### 3.1. Patients’ Characteristics

A total of 91 patients met the inclusion criteria (*RUNX1+*, n = 33 and *RUNX1−,* n = 58) and entered into the present analysis of this study. Complete *RUNX1* mutational data were available at diagnosis for all patients. For the whole cohort, the median follow-up was 20.1 (16–38.3) months, the median age was 38 (range 6–64) years, 33 (35.9%) patients were female. AML was classified as de novo disease in 84.8 and 89.7% of *RUNX1+* and *RUNX1−* patients, respectively. Most patient, disease, and transplant characteristics were equally distributed between the two cohorts (Table 1). At two years, eight patients had died. AML was the most frequent cause of death, accounting for 50% of all fatalities, followed by infections (37.5%) and GvHD (12.5%).

An imbalance in the frequency of SRSF-2, IDH2, and TP53 mutations was observed (Table 2). SRSF-2 mutation was detected in 18.8% of *RUNX1+* patients but only 1.8% of *RUNX1−* patients (*p* < 0.05). In contrast, we did not observe significant frequency differences between *ASXL1* (15.2 vs. 13.8%, *p* = 0.859) and *NPM1* (3.0 vs. 13.8%, *p* = 0.198). *DNMT3A* and *FLT3-ITD* were observed at a similar frequency (15.2 vs. 15.5%, *p* = 0.963; 27.3 vs. 19%, *p* = 0.358, respectively) in both groups.

### 3.2. Transplantation Outcomes and Univariate Analysis

**Figure 1:**
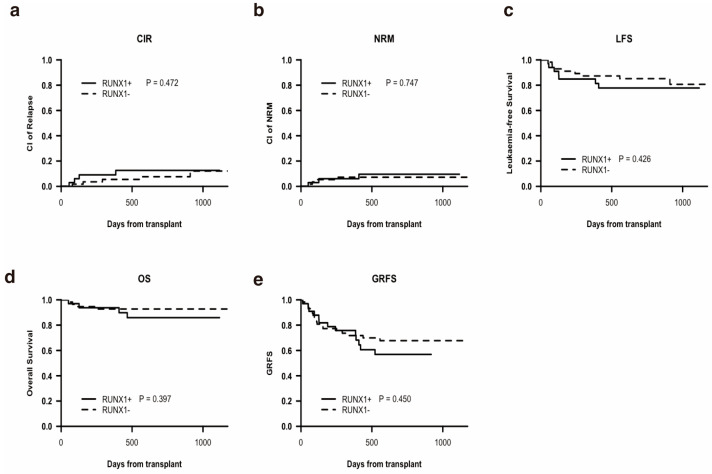
Outcomes after allo-HSCT according to mutation status (n = 91). (**a**) Cumulative relapse incidence (CIR), (**b**) non-relapse mortality (NRM), (**c**) leukemia-free survival (LFS), (**d**) overall survival (OS), (**e**) GvHD-free/relapse-free survival (GRFS).

The 2-year CIR rate after allo-HSCT for the study cohort was 9.5% (95% CI 3.2–15.8). Figure 1a demonstrates no significant difference in CIR between RUNX1+ and RUNX1− groups (*p* = 0.472; 2-year CIR: 12.6% vs. 7.6%). Univariate analysis revealed NRAS mutations as a contributor to higher CIR, with no other factors showing significant correlations (Supplementary Table S1).

The 2-year NRM rate was 8.0% (95% CI 2.3–13.7). As shown in Figure 1b, NRM rates were comparable between RUNX1+ and RUNX1− groups (*p* = 0.747; 2-year NRM: 9.6% vs. 7.2%). Univariate analysis indicated that allo-HSCT in first complete remission (CR1) status and low HCT-CI score correlated with lower NRM, while other variables were not associated with NRM (Supplementary Table S1).

The 2-year LFS rate was 82.5% (95% CI 74.8–91.1). Figure 1c shows no notable difference in LFS between RUNX1+ and RUNX1− groups (*p* = 0.426; 2-year LFS: 77.8% vs. 85.2%). Univariate analyses identified CR1 status at transplant and age <38 years as predictors of extended LFS, whereas NRAS mutations were linked to lower LFS. Other factors showed no effect (Supplementary Table S1).

The 2-year OS rate for the cohort was 90.5% (95% CI 84.4–97.0), with infections being the leading cause of mortality, followed by relapse. The RUNX1+ and RUNX1− groups had similar 2-year OS rates (85.9% vs. 92.7%; *p* = 0.397), as depicted in Figure 1d. Univariate analyses confirmed that low HCT-CI score and transplantation in CR1 status were associated with longer OS (Supplementary Table S1).

The 2-year GRFS rate was 64.1% (95% CI 54.7–75.2). The RUNX1+ and RUNX1− groups exhibited comparable GRFS rates (56.8% vs. 67.7%; *p* = 0.450), as presented in Figure 1e. Univariate analysis highlighted transplantation in non-CR1 status and FLT3-ITD mutations as major contributors to inferior GRFS (Supplementary Table S1).

The median (range) infused CD34+ stem cell and mononuclear cell doses were 3.41 (0.75–12.69) × 10^6^/kg and 5.23 (9.45–16.1) × 10^8^/kg, respectively. In the entire cohort, all patients attained neutrophil recovery, with a median time to platelet and neutrophil engraftment after Haplo-HSCT of 14 and 13 days, respectively. Primary platelet engraftment failure occurred in three patients; successful platelet recovery was not observed in three patients who died after allo-HSCT, and the remaining 93.4% of patients achieved platelet recovery. The two groups had no significant difference in time to platelet or neutrophil engraftment (*p* = 0.394 and 0.488, respectively). The 100-day cumulative incidence of Grade II-IV aGVHD in the entire cohort was 25.4% (95% CI 16.4–34.4). The 100-day cumulative incidences of Grade II–IV aGVHD were 18.2% and 24.1% in the *RUNX1+* and *RUNX1−* (*p* = 0.901). The 2-year cumulative incidence of cGVHD in the entire cohort was 49.7% (95% CI 38.4-61.1). Two patients in the whole cohort developed severe cGVHD after Haplo-HSCT. The 2-year incidence of cGVHD was similar in the *RUNX1+* and *RUNX1−* groups (48.1% and 50.2%, respectively, *p* = 0.670). Univariate analysis found no variables associated with Grade II-IV aGVHD, and patients who received Haplo-HSCT had lower cGVHD (Supplementary Table S1).

When the analysis was limited to 73 patients (25 *RUNX1+* and 48 *RUNX1−*) receiving Haplo-HSCT, no influence of the *RUNX1* mutation on any outcome parameter could be demonstrated (Supplementary Tables S2 and S3, Supplementary Figure S1). Interestingly, in the subgroup of Haploidentical recipients, the effects of high HCT-CI scores on NRM and OS and the effects of *NRAS* gene mutations on CIR and LFS were not observed in univariate analyses. Similarly, no impact of *RUNX1* mutations on any outcome parameter was observed when the analysis was restricted to specific subgroups: 18 patients who underwent HLA-matched sibling HSCT, 79 patients (87%) with de novo AML, or 55 patients (76%) with intermediate-risk cytogenetics (Supplementary Table S4, Supplementary Figures S2 and S3).

## 4. Discussion

This study compared the clinical outcomes between the *RUNX1+* and *RUNX1−* groups after allo-HSCT, focusing on Haplo-HSCT. There was no notable difference in 2-year clinical outcomes between the RUNX1+ and RUNX1− groups.The information on the role of the *RUNX1* mutation on outcomes after allo-HSCT (especially Haplo-HSCT) is scarce. Therefore, to address this gap, we performed a retrospective analysis of previous case data on *RUNX1* mutation status in AML patients to further explore the impact of *RUNX1* on patient prognosis. 

According to previous reports [1,6], the mutation rate of the *RUNX1* gene is approximately 10% in de novo AML. In our cohort, the incidence was higher at 36%, as we included only patients with complete *RUNX1* mutation data at initial diagnosis. Unlike previous studies, which primarily included older patients, our study focused on younger patients with de novo AML, with a median age of 38 (6–64). In a 2021 study by Waidhauser et al. [16], the median age of AML patients with *RUNX1+* was 57.4 years, and 2-year OS and LFS after transplantation was 66.6% (95% CI: 62.4–70.8) and 61% (95% CI: 62.4-70.8). In our cohort, AML patients with *RUNX1+* had better survival outcomes, which may be attributed to the younger age of our patients and the higher proportion who underwent Haplo-HSCT. Specifically, 80.2% of patients in our cohort underwent Haplo-HSCT, compared to only 8.0% in Waidhauser’s study. Compared with Waidhauser et al., the subgroup of patients undergoing Haplo-HSCT in our study exhibited superior OS (85.8% vs. 67.7%) and LFS (82.3% vs. 61.1%). A comparable trend was observed when our results were contrasted with those of Chan et al. [14]. Additionally, differences in the conditioning regimen (ATG vs. TBI) and GVHD prophylaxis (MTX + CSA + MMF vs. PTCy + calcineurin inhibitor + MMF) may also contribute to the observed variations between the two studies. The P-value for the prognostic difference between *RUNX1+* and *RUNX1−* was not significant in the subgroup of HLA-matched sibling transplants. However, this lack of statistical significance may be attributed to the small sample size of the cohort with a notable trend in survival outcomes: the 2-year LFS was 62.5% for *RUNX1+* versus 88.9% for *RUNX1−*, and the 2-year OS was 87.5% compared to 100.0%, respectively. In our study, patients undergoing Haploidentical transplantation demonstrated higher LFS (82.3% vs. 62.5%) compared to those receiving HLA-matched transplantation. By contrast, Della Porta et al. [15], whose study focused solely on HLA-matched transplantation, reported significantly lower survival rates in the RUNX1-mutated group. The observed outcome differences may be attributed to the potent GVL effects associated with Haplo-HSCT, suggesting that patients with RUNX1+ may derive at least partial clinical benefit from this approach. 

We performed a comparative analysis between our findings and previous studies investigating the prognostic significance of RUNX1 mutations in AML patients undergoing allo-HSCT, aiming to identify key factors that may account for the observed discrepancies. In our cohort, the presence of RUNX1 mutations was not associated with a significantly adverse impact on survival. Similarly, Chou et al. [17] identified RUNX1 mutations as an independent favorable prognostic factor in AML patients receiving allo-HSCT, with improved survival outcomes, which aligns partially with our observations. Both studies were retrospective in design; however, Chou’s analysis included only 12 RUNX1+ patients who underwent allo-HSCT, potentially involving atypical cases. Furthermore, differences in treatment strategies over the 12-year study period, along with a substantial discrepancy in the use of Haplo-HSCT (9% in Chou’s cohort vs. 80.2% in ours), may account for the variations in outcomes. Conversely, Chan et al. [14] reported inferior overall survival and higher relapse rates in AML patients with RUNX1 mutations following allo-HSCT, findings that contradict our results. In their cohort, 13 of 28 patients (46.4%) were aged ≥60 years, compared to only 1 of 33 patients (3%) in our study—an age distribution difference that may have significantly influenced survival outcomes. Additionally, the proportion of patients receiving Haplo-HSCT was considerably lower in Chan’s study (28.6%) than in ours (80.2%), which may further contribute to the differing prognostic impacts observed across studies.

In patients undergoing allo-HSCT for sAML, Della Porta et al. reported that *RUNX1* mutations were associated with poor prognosis [15]. In contrast, our analysis did not reveal any negative impact of *RUNX1* mutations on post-transplant outcomes. This finding was consistent across the entire cohort and in focused analyses of patients with de novo AML (comprising over 91% of the cohort) and those with intermediate-risk cytogenetics [16]. The discrepancy between our results and those of Della Porta may be attributed to several factors, including the inclusion of MDS and sAML patients in their study, the absence of CR status analysis at the time of transplantation, and differences in treatment protocols. Furthermore, the different proportions of patients receiving Haplo-HSCT may also account for the variation.

The coexistence of *RUNX1* and *ASXL1* mutations has been reported as a solid adverse prognostic factor in previous studies [36,37]. Della Porta et al. [15] demonstrated that ASXL1 mutations are independently associated with poorer outcomes and reduced survival in patients with MDS/AML following allo-HSCT. However, contrary to expectations, a higher incidence of *ASXL1* mutations was not observed in patients with *RUNX1+*. This may be due to the small sample size. In our study, *ASXL1* as a single factor did not have any influence on outcome in univariate analysis, nor did the different combinations of *RUNX1* and *ASXL1* mutational status (Supplementary Table S5). This finding aligns with an extensive study by J. Waidhauser et al., who could not detect a functional interaction between the two mutations. Similarly, Schnittger et al. [6] observed that different combinations of RUNX1 and ASXL1 mutation status had no significant impact on clinical outcomes. Our data indicate that Haplo-HSCT may enhance the prognosis of patients with co-occurring RUNX1 and ASXL1 mutations (RUNX1+/ASXL1+).

The 2022 International Consensus Classification (ICC) categorized AML harboring nine sAML-type (secondary AML-type) mutations, including RUNX1, as AML with myelodysplasia-related (MDS-R) gene mutations, which are closely associated with sAML and poorer prognosis [38]. Tsai et al. [39] conducted an exploratory analysis of cohorts with available data on MDS-R co-mutations to assess their impact on survival in AML patients. The study revealed a significant interaction between these mutations, resulting in an inferior prognosis. Specifically, MDS-R mutations markedly negatively affected OS and EFS in younger patients undergoing allo-HSCT during the first remission. Gardin et al. [40] found that allo-HSCT may counteract the adverse prognostic effects in AML patients with either: (1) ELN-2017 intermediate-risk carrying MDS-R mutations, or (2) ELN-2017 adverse-risk classification. However, no significant prognostic benefit was observed for patients with ELN-2017 favorable-risk or ELN-2017 intermediate-risk AML (without MDS-R mutations).

Interestingly, we did not observe any prognostic effect of MDS-R mutations in our cohort (Supplementary Table S5). One possible explanation is that the study by Tsai spanned nearly 30 years, during which time advances in treatment regimens and concepts, such as introducing the Beijing Protocol, significantly improved patient outcomes. Additionally, our study had a smaller sample size and a shorter observation period, which may have limited our ability to capture the full impact of MDS-R mutations on long-term prognosis.

A key question arising from these findings is which subgroups of AML patients are most likely to benefit from allo-HSCT. Our preliminary data suggest that AML patients with RUNX1mt who were in CR1 at the time of transplantation and had a low HCT-CI score experienced better outcomes. This underscores the importance of early transplantation in improving prognosis. Furthermore, our results indicate that allo-HSCT, especially Haplo-HSCT, may help offset the poor prognosis in patients with co-occurring RUNX1+/ASXL1+ or MDS-R gene mutations.

This study has several limitations. It is a single-center, retrospective analysis, limiting the generalizability of our findings. The small sample size also affects the study’s statistical power, and the groups are too small to allow for a reliable multivariate analysis. Furthermore, extended follow-up is necessary to evaluate the long-term post-transplant survival of AML patients harboring RUNX1 mutations. Finally, the relatively young median age of the cohort limits our ability to evaluate prognostic differences between younger and older patients. The limitations of our study preclude its findings from fully representing diverse patient populations and comprehensively evaluating the impact of RUNX1 mutations on long-term survival. Conflicting evidence from prior studies, combined with their methodological constraints, impedes a thorough understanding of the prognostic role of RUNX1 mutations. We therefore advocate for larger-scale, prospective, multicenter studies to further elucidate this issue.

## 5. Conclusions

In conclusion, since *RUNX1* mutations were not linked to worse outcomes in our study, our findings suggest that allo-HSCT, particularly Haplo-HSCT, may improve the prognosis of AML patients with *RUNX1* mutations. Further validation in larger cohorts and comprehensive molecular profiling is necessary to explore the interactions between different mutations and their influence on post-transplant outcomes.

## Figures and Tables

**Table 1:** Patient and transplantation characteristics.

	All	RUNX1+	RUNX1−	*p*
**Characteristics**	n = 91	n = 33	n = 58	
**Age at HSCT, years, median (range)**	38 (6–64)	44 (14–60)	35.5 (6–64)	0.086
**Sex, n (%)**				0.639
Male	58(63.7)	20 (60.6)	38 (65.5)	
female	33(36.3)	13 (39.4)	20 (34.5)	
**Donor type**				0.42
haplo	73 (80.2)	25 (75.8)	48 (82.8)	
MSD/MUD	18 (19.8)	8 (24.2)	10 (17.2)	
**Transplantation, n (%)**				0.783
CR1	65 (71.4)	23 (69.7)	42 (72.4)	
Non-CR1	26 (28.6)	10 (30.3)	16 (27.6)	
**Donor-recipient blood type match, n (%)**				0.112
Matched	54 (59.3)	16 (48.5)	38 (65.5)	
Mismatched	37 (40.3)	17 (51.5)	20 (34.5)	
**Time from diagnosis to HSCT, median, range** (range)	211 (62–1079)	29 (87.9)	220.5 (62–679)	0.509
**Sex match, n (%)**				0.456
Male recipient–female donor	16 (17.6)	4 (12.1)	12 (20.7)	
Any other	75 (82.4)	29 (87.9)	46 (79.3)	
**HCT-CI, n (%)**				0.487
0–2	81 (89.0)	28 (84.8)	53 (91.4)	
>3	10 (11.0)	5 (15.2)	5 (8.6)	
**Pre-MRD, n(%)**				0.358
Positive	20 (30.0)	9 (27.3)	11 (19)	
Negative	71 (70.0)	24 (72.7)	47 (81)	
**CK, n (%)**				0.745
NO	74 (81.3)	25 (83.3)	49 (87.5)	
CK	13 (14.3)	5 (16.7)	7 (12.5)	
Missing	NA = 5	NA = 3	NA = 2	
**HLA, n (%)**				0.593
3/6, 5/10	53 (58.2)	17 (51.5)	36 (62.1)	
4/6, 6/10, 7/10, 8/10	20 (30.0)	8 (24.2)	12 (20.7)	
6/6, 10/10	18 (11.8)	8 (24.2)	10 (17.2)	
**Disease type, n%**				0.519
denovo	80 (87.9)	28 (84.8)	52 (89.7)	
secondary	11 (12.1)	5 (15.2)	6 (10.3)	
**Cytogenetic risk-2022ELN**				0.161
favorable	9 (9.9)	1 (3.3)	8 (14.3)	
intermediate	63 (69.2)	22 (73.3)	41 (73.2)	
poor	14 (15.4)	7 (23.3)	7 (12.5)	
Missing	NA = 5	NA = 3	NA = 2	

Abbreviations: MSD, matched sibling donor; MUD, matched unrelated donor; CK, complex karyotype; CR1, first complete remission; HCT-CI, Haematopoietic Cell Transplant Comorbidity Index; HLA, human leucocyte antigen; HSCT, hematopoietic stem cell transplant; MRD, minimal residual disease.

**Table 2:** Comparison of genetic alterations between the RUNX1+ and RUNX1− groups.

Common Molecular Mutation (%)	All	RUNX1+	RUNX1−	*p*
**KMT2A-PTD**				0.198
positive	9 (9.9)	1 (3.0)	8 (13.8)	
negative	82 (90.1)	32 (97.0)	50 (86.2)	
**FLT3-ITD**				0.358
positive	20 (30.0)	9 (27.3)	11 (19)	
negative	71 (70.0)	24 (72.7)	47 (81)	
**ASXL1**				0.859
positive	13 (14.3)	5 (15.2)	8 (13.8)	
negative	78 (85.7)	28 (84.8)	50 (86.2)	
**CEBPA**				0.198
positive	9 (9.9)	1 (3.0)	8 (13.8)	
negative	82 (90.1)	32 (97.0)	50 (87.2)	
**NRAS**				0.577
positive	9 (9.9)	2 (6.1)	7 (12.1)	
negative	82 (90.1)	31 (93.9)	51 (87.9)	
**BCORL1**				0.264
positive	4 (4.4)	3 (9.1)	1 (1.7)	
negative	87 (95.6)	30 (90.9)	57 (98.3)	
**SRSF-2**				0.017
positive	7 (7.7)	6 (18.8)	1 (1.8)	
negative	80 (87.9)	26 (81.2)	54 (98.2)	
Missing	NA = 4	NA = 1	NA = 3	
**DNMT3A**				0.963
positive	14 (15.4)	5 (15.2)	9 (15.5)	
negative	77 (84.6)	28 (84.8)	49 (84.5)	
**IDH1**				>0.99
positive	6 (6.6)	2 (6.1)	4 (6.9)	
negative	85 (93.4)	31 (93.9)	54 (93.1)	
**IDH2**				0.041
positive	6 (6.6)	5 (15.2)	1 (1.7)	
negative	85 (93.4)	28 (84.8)	57 (98.3)	
**NPM1**				0.198
positive	9 (9.9)	1 (3)	8 (13.8)	
negative	82 (90.1)	32 (97)	50 (86.2)	
**TP53**				0.041
positive	6 (6.6)	5 (15.2)	1 (1.7)	
negative	85 (93.4)	28 (84.8)	57 (98.3)	

## Data Availability

The original contributions presented in this study are included in the article/supplementary material. Further inquiries can be directed to the corresponding author.

## Supplementary Materials

The following supporting information can be downloaded at: https://www.mdpi.com/article/10.3390/curroncol32060294/s1, Figure S1: Outcome of 73 patients with or without RUNX1 gene mutation received Haplo-HSCT; Figure S2: Outcome of 79 patients with de novo AML with or without RUNX1 gene mutation; Figure S3: Outcome of 55 patients with AML and intermediate risk cytogenetics with or without RUNX1 gene mutation; Table S1: Univariate analysis of OS, LFS, CIR, NRM, aGVHD, cGVHD and GRFS; Table S2: Patient and transplantation characteristics of the Haplo-HSCT cohort (n = 73); Table S3: Univariate analysis of OS, LFS, CIR, NRM, aGVHD, cGVHD and GRFS in the Haplo-HSCT cohort; Table S4: Outcome among patients with de novo AML (n = 79) and patients with intermediate-risk cytogenetics (n = 55), and patients who received HLA-matched sibling HSCT(n = 18) as of RUNX1 mutational status; Table S5: Exploratory analysis of further genetically defined subgroups.

## Abbreviations

The following abbreviations are used in this manuscript:

CR	Complete remission
LFS	Leukemia-free survival
allo-HSCT	Allogeneic hematopoietic stem cell transplantation
Haplo-HSCT	Haploidentical HSCT
NRM	Non-relapse mortality
CIR	Cumulative incidence of relapse
GRFS	GvHD-free/relapse-free survival
AML	Acute myeloid leukemia
CK	Complex karyotype
HCT-CI	Hematopoietic Cell Transplant Comorbidity Index
HLA	Human leucocyte antigen
MRD	Minimal residual disease
