# Supplementary material for: Clinical Effects of RUNX1 Mutations on the Outcomes of Patients with Acute Myeloid Leukemia Treated with Allogeneic Hematopoietic Stem-Cell Transplantation
Source: Curr Oncol. 2025 May 22;32(6):294. doi: 10.3390/curroncol32060294 (PMC12192008; doi:10.3390/curroncol32060294)
Supplement: Supplementary file 1 [file curroncol-32-00294-s001.zip › curroncol-3582652-supplementary.pdf]

Supplementary Materials:

Supplementary Figure 1: Outcome of 73 patients with or without RUNX1 gene mutation received Haplo-HSCT

a) cumulative relapse incidence (CIR), b) non-relapse mortality (NRM), c) leukemia-free survival (LFS), d) overall survival (OS), e) GvHD-free/relapse-free survival (GRFS)

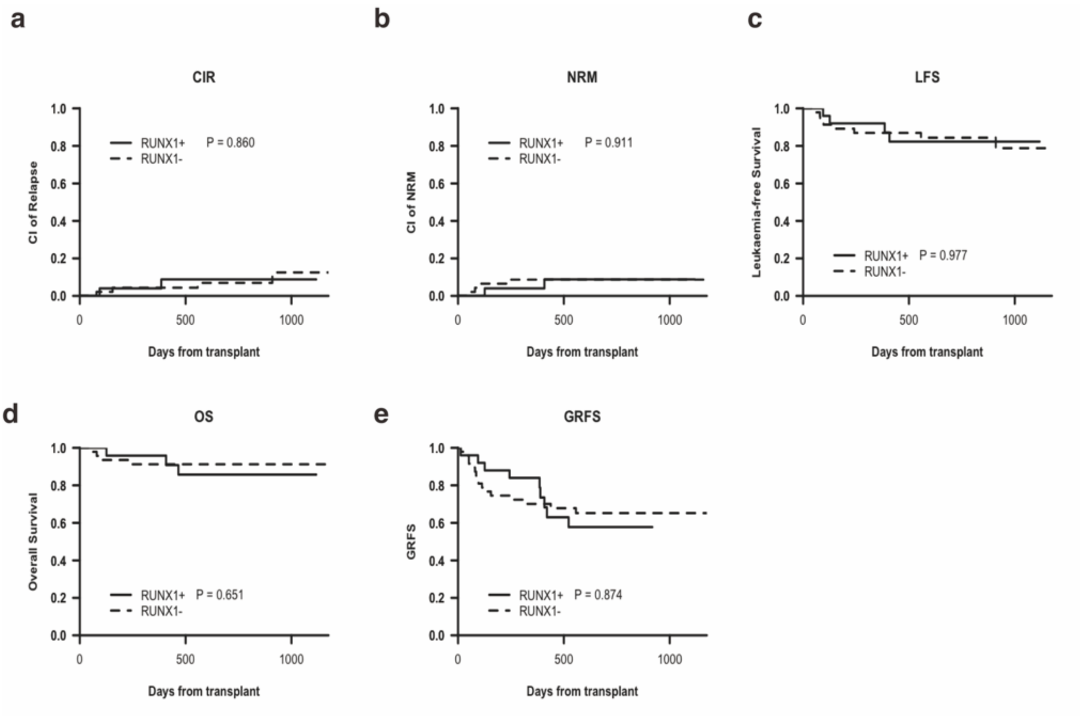

Supplementary Figure 2: Outcome of 79 patients with de novo AML with or without RUNX1 gene mutation

a) cumulative relapse incidence (CIR), b) non-relapse mortality (NRM), c) leukemia-free survival (LFS), d) overall survival (OS), e) GvHD-free/relapse-free survival (GRFS)

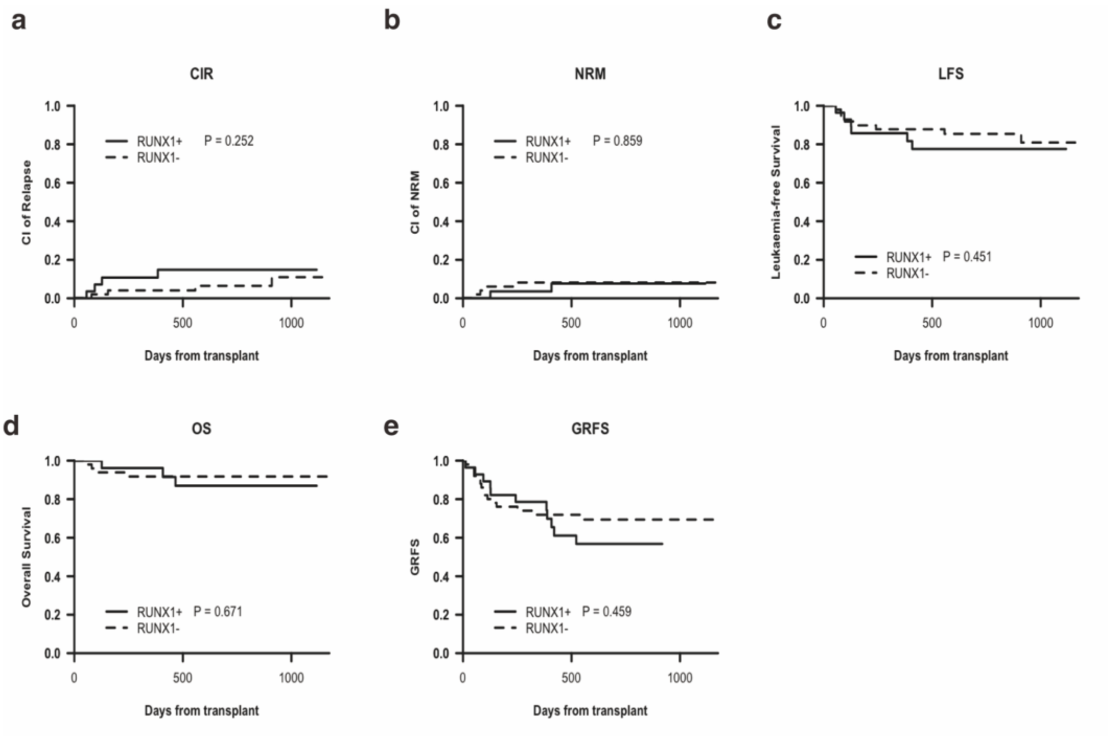

Supplementary Figure 3: Outcome of 55 patients with AML and intermediate risk cytogenetics with or without RUNX1 gene mutation

a) cumulative relapse incidence (CIR), b) non-relapse mortality (NRM), c) leukemia-free survival (LFS), d) overall survival (OS), e) GvHD-free/relapse-free survival (GRFS)

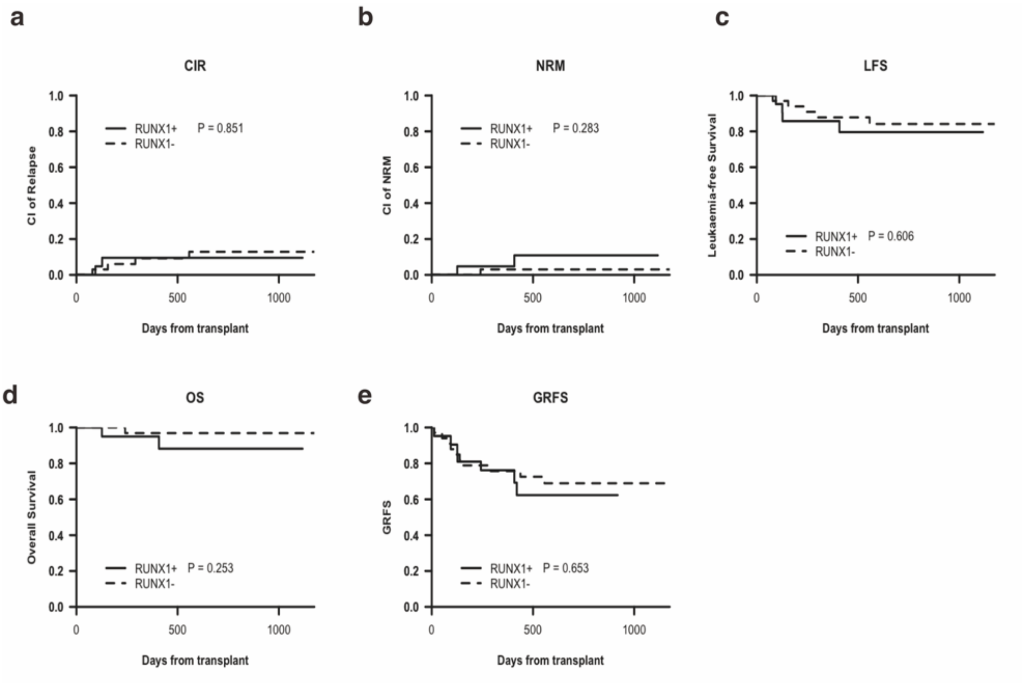

Supplementary Table 1. Univariate analysis of OS, LFS, CIR, NRM, aGVHD, cGVHD and GRFS

	CIR	P	NRM	P	LFS	P	OS	P	GRFS	P	Grade II–IV aGVHD	P	cGVHD	P
	2years, % 95%CI		2years, % 95%CI		2years, % 95%CI		2years, % 95%CI		2years, % 95%CI		2years, % 95%CI		2years, % 95%CI	
**RUNX1 status**		0.472		0.747		0..426		0.397		0.450		0.901		0.670
RUNX1-	7.6(0.3-14.9)		7.2(0.3-14.1)		85.2(76.1-95.2)		92.7(86.1-99.9)		67.7(56.5-81.3)		24.1(13.0-35.3)		50.2(36.0-64.3)	
RUNX1+	12.6(0.8-24.5)		9.6(0-20.2)		77.8(64.5-93.9)		85.9(73.9-99.9)		56.8(41.3-78.1)		18.2(4.8-31.6)		48.1(29.1-67.0)	
**Age at HSCT**		0.062		0.273		0.025		0.447		0.109		0.980		0.397
<38	4.8(0-11.5)		4.7(0-11.2)		90.5(82-99.8)		92.8(85.2-100.0)		74.0(61.8-88.5)		25.0(12.0-38.0)		44.4(28.4-60.4)	
≥38	14.2(3.4-25.0)		11.1(1.8-20.5)		74.7(62.7-89.0)		88.4(79.3-98.6)		54.9(41.7-72.1)		19.1(7.8-30.5)		55.6(39.2-72.1)	
**Sex**		0.355		0.672		0.315		0.499		0.090		0.562		0.792
female	6.5(0-15.3)		6.4(0-15.0)		87.2(76.2-99.8)		93.5(85.3-100.0)		77.8(64.5-93.8)		27.3(11.8-42.7)		48(28.7-67.2)	
Male	11.2(2.6-19.7)		9.0(1.4-16.6)		79.8(69.8-91.3)		88.7(80.5-97.7)		56.3(44.3-71.4)		19.0(8.8-29.2)		51.1(36.7-65.5)	
**Donor type**		0.296		0.720		0.548		0.630		0.892		0.717		0.020
haplo	7.5(1.1-14.0)		8.6(2-15.2)		83.9(75.5-93.1)		89.6(82.6-97.2)		63.3(52.7-76.1)		24.7(14.7-34.6)		45.4(32.6-58.2)	
MSD/MUD	17.4(0-36.0)		5.6(0-16.4)		77.0(59.6-99.6)		94.4(84.4-100.0)		66.7(48.1-92.4)		11.1(0-26.1)		69.3(45.0-93.5)	
**Transplantation**		0.180		<0.001		<0.001		<0.001		0.009		0.350		0.721
CR1	6.8(0.3-13.2)		1.7(0-5.0)		91.6(84.7-99.0)		98.3(94.9-100.0)		70.8(60.2-83.2)		18.5(8.9-28.0)		51.1(37.8-64.4)	
Non-CR1	16.6(1.2-31.9)		24.0(6.8-41.2)		59.4(42.8-82.5)		70.2(53.8-91.6)		46.7(30.4-71.8)		30.8(12.6-48.9)		46.9(24.1-69.7)	

	CIR	P	NRM	P	LFS	P	OS	P	GRFS	P	Grade II–IV aGVHD	P	cGVHD	P
	2years, % 95%CI		2years, % 95%CI		2years, % 95%CI		2years, % 95%CI		2years, % 95%CI		2years, % 95%CI		2years, % 95%CI	
**Donor-recipient blood type match**		0.762		0.388		0.396		0.564		0.728		0.231		0.942
Matched	8.1(0.4-15.8)		5.7(0-12.1)		86.2(77.2-96.3)		92.0(84.7-99.9)		66.6(54.7-81.1)		25.9(14.1-37.7)		48.8(33.5-64.1)	
Mismatched	11.5(0.7-22.3)		11.2(0.7-21.7)		77.3(64.6-92.5)		88.2(77.9-99.8)		61.2(47.2-79.5)		16.2(4.2-28.3)		50.8(33.3-68.3)	
**Time from diagnosis to HSCT**		0.647		0.689		0.526		0.940		0.935		0.858		0.797
<211	9.3(0.5-18.1)		6.8(0-14.4)		83.9(73.6-95.6)		90.7(82.3-99.8)		63.4(50.6-79.5)		22.2(9.9-34.5)		47.7(32.1-63.2)	
≥211	9.7(0.4-18.9)		9.2(0.5-17.8)		81.2(70.2-93.9)		90.6(82.2-99.8)		65.0(52.0-81.3)		21.7(9.7-33.8)		51.8(35.0-68.6)	
**Sex match**		0.570		0.832		0.761		0.753		0.381		0.905		0.096
Any other	8.7(2-15.4)		8.3(1.9-14.7)		83.0(74.7-92.3)		93.1(87.5-99.1)		66.2(55.9-78.3)		22.7(12.7-32.6)		37.1(25.6-48.6)	
Male recipient–female donor	13.3(0-31.2)		6.7(0-19.8)		80.0(62.1-100.0)		93.3(81.5-100.0)		55.0(35.0-86.5)		18.8(0-41.3)		44.7(15.2-74.2)	
**HCT-CI**		0.300		0.006		0.189		0.010		0.318		0.748		0.228
0-2	10.7(3.6-17.7)		5.2(0.2-10.2)		84.1(76.2-92.8)		93.1(87.5-99.2)		66.1(56.3-77.8)		22.2(13.1-31.3)		42.2(30.7-53.8)	
>3	0		30.0(0-60.2)		70.0(46.7-100.0)		70.0(46.7-100.0)		50.0(26.9-92.9)		20.0(0-46.1)		10.0(0-30.1)	
**Pre-MRD**		0.396		0.172		0.067		0.210		0.051		0.998		0.534
Negative	7.9(1.2-14.6)		6.0(0.2-11.8)		86.1(78.0-95.0)		92.3(86.0-99.0)		68.3(57.9-80.6)		21.1(11.6-30.7)		48.4(35.7-61.1)	
Positive	15.0(0-31.1)		15.0(0-31.1)		70.0(52.5-93.3)		84.4(69.6-100.0)		49.5(31.7-77.4)		25.0(5.5-44.5)		55.2(28.9-81.5)	
**CK**		0.731		0.269		0.746		0.373		0.401		0.220		0.770
NO	10.3(3-17.7)		7.2(1.1-13.4)		82.4(73.8-92)		92.6(86.6-99.1)		66.5(56.1-78.8)		21.6(12.2-31.1)		49.5(36.6-62.4)	
YES	0		15.4(0-35.8)		84.6(67.1-100.0)		83.3(64.7-100.0)		53.8(32.6-89.1)		33.3(5.1-61.5)		55.6(23.3-87.8)	

	CIR	P	NRM	P	LFS	P	OS	P	GRFS	P	Grade II–IV aGVHD	P	cGVHD	P
	2years, % 95%CI		2years, % 95%CI		2years, % 95%CI		2years, % 95%CI		2years, % 95%CI		2years, % 95%CI		2years, % 95%CI	
**Disease type**		0.950		0.884		0.540		0.929		0.904		0.966		0.573
denovo	9.4(2.7-16.2)		7.9(1.8-14.0)		82.7(74.5-91.7)		90.5(84-97.5)		65.3(55.3-77.2)		23.8(14.4-33.1)		45.3(33.7-57.0)	
secondary	10.2(0-30.2)		8.3(0-24.7)		81.5(61.1-100.0)		90.9(75.4-100.0)		56.2(33.6-94.3)		9.1(0-26.9)		53.0(17.1-89.0)	
**Cytogenetic risk**		0.587		0.520		0.636		0.603		0.196		0.956		0.303
favorable	0		12.5(0-37.0)		87.5(67.3-100.0)		67.3(67.3-100.0)		77.8(54.9-100.0)		22.2(0-51.2)		70.8(27.6-100.0)	
intermediate	10.2(2.3-18.1)		6.6(0.3-12.9)		83.2(74.1-93.3)		93.2(86.9-99.9)		67.9(56.9-81.1)		23.8(13.2-34.4)		44.8(31.5-58.1)	
poor	7.1(0-21.2)		14.3(0-33.3)		78.6(59.8-100.0)		85.7(69.2-100.0)		42.9(23.4-78.5)		21.4(0-43.8)		51.4(20.8-82.0)	
**NRAS**		0.014		0.716		0.024		0.786		0.207		0.338		0.250
negative	6.4(0.9-11.9)		7.7(1.7-13.6)		85.9(78.5-94.0)		90.7(84.4-97.5)		67.1(57.4-78.3)		23.2(14.0-32.4)		44.2(32.7-55.7)	
positive	35.6(0-71.4)		11.1(0-33.1)		28.2(28.2-100.0)		87.5(67.3-100.0)		34.6(12.2-97.8)		11.1(0-32.9)		75.0(38.4-100.0)	
**ASXL1**		0.557		0.998		0.646		0.8850		0.762		0.801		0.058
negative	9.8(2.8-16.8)		8.1(1.8-14.3)		82.1(73.7-91.5)		90.2(83.5-97.4)		65.0(54.9-76.9)		23.1(13.7-32.5)		45.7(33.3-58.0)	
positive	7.7(0-22.8)		7.7(0-22.8)		84.6(67.1-100.0)		92.3(78.9-100.0)		59.8(37.8-94.7)		15.4(0-35.9)		73.1(44.0-100.0)	
**DNMT3A**		0.099		0.892		0.387		0.853		0.959		0.296		0.234
negative	7.1(1.0-13.2)		8.1(1.8-14.4)		84.7(76.8-93.5)		90.3(83.7-97.4)		64.4(54.2-76.5)		19.5(10.6-28.4)		45.3(33.4-57.2)	
positive	22.1(0-45.4)		7.1(0-21.2)		70.7(50.2-99.6)		91.7(77.3-100.0)		62.9(41.6-95.0)		35.7(9.4-62.0)		91.7(38.2-100.0)	
**SRSF-2**		0.621		0.548		0.216		0.706		0.568		0.918		0.999
negative	7.9(1.8-14.1)		7.8(1.8-13.9)		84.2(76.4-92.9)		90.5(84.0-97.5)		66.2(56.4-77.8)		23.8(14.4-33.1)		45.8(34.1-57.6)	
positive	14.3(0-42.7)		14.3(0-42.3)		71.4(44.7-100.0)		85.7(63.3-100.0)		53.6(25.7-100.0)		14.3(0-42.3)		47.6(0.7-94.6)	
**FLT3-ITD**		0.393		0.711		0.341		0.832		0.023		0.940		0.630
negative	7.7(1.1-14.4)		7.4(1.1-13.7)		84.9(76.6-94.0)		90.9(84.1-98.1)		70.3(60.1-82.2)		21.1(11.6-30.7)		50.7(37.9-63.5)	
positive	15.0(0-31.1)		10.0(0-23.5)		75.0(58.2-96.6)		89.2(76.0-100.0)		42.4(24.9-72.3)		25.0(5.5-44.5)		46.1(20.8-71.4)	
**NPM1**		0.979		0.101		0.250		0.156		0.223		0.172		0.404
negative	9.2(2.6-15.8)		6.4(0.9-11.9)		84.4(76.6-93.0)		92.0(86.0-98.4)		66.6(56.8-78.1)		19.5(10.9-28.1)		49.3(37.5-61.1)	
positive	11.1(0-33.3)		22.2(0-51.2)		66.7(42.0-100.0)		77.8(54.9-100.0)		44.4(21.4-92.3)		44.4(9.4-79.5)		48.1(8.1-88.2)	

Abbreviations: MSD, matched sibling donor; MUD, matched unrelated donor; CK, complex karyotype; CIR, the cumulative incidence of relapse; GRFS, GvHD--free/relapse--free survival; (a)(c) GvHD, (acute) (chronic) graft--versus--host disease; HCT-CI, Hematopoietic Cell Transplant Comorbidity Index; HSCT, hematopoietic stem cell transplant; LFS, leukemia--free survival; MRD, minimal residual disease; NRM, non--relapse mortality rate; OS, overall survival.

Supplementary Table 2. Patient and transplantation characteristics of the Haplo-HSCT cohort (n=73)

	All	RUNX1+	RUNX1-	P
Characteristics	n=73	n=25	n=48	
Age at HSCT, years, median (range)	35(6-64)	39(14-60)	34.5(6-64)	0.399
Sex, n (%)				0.572
Male	47(64.4)	10(40.0)	16(33.3)	
Female	26(35.6)	15(60.0)	32(66.7)	
Transplantation, n (%)				0.802
Non-CR1	22(30.1)	8(32.0)	14(29.2)	
CR1	51(69.9)	17(68.0)	34(70.8)	
Donor-relationship				0.639
Cousions or Parents	53(72.6)	6(24.0)	14(29.2)	
Siblings	20(27.4)	19(76.0)	34(70.8)	
Donor-recipient blood type match, n (%)				0.297
Mismatched	29(39.7)	12(48.0)	17(35.4)	
Matched	44(60.3)	13(52.0)	31(64.6)	
Time from diagnosis to HSCT, median, range (range)	211(62-1079)	201(80-1079)	220.5(62-679)	0.457
Sex match, n (%)				0.082
Male recipient–female donor	12(16.4)	1(4.0)	11(22.9)	
Any other	61(84.6)	24(96.0)	37(77.1)	
HCT-CI, n (%)				0.482
>3	9(12.3)	4(16.0)	5(10.4)	
0-2	64(87.7)	21(84.0)	43(89.6)	
Pre-MRD, n (%)				0.898
Positive	14(19.2)	5(20.0)	9(18.3)	
Negative	59(80.8)	20(80.0)	39(81.3)	
CK, n (%)				0.656
CK	6(8.8)	1(4.5)	5(10.9)	
NO	62(91.2)	21(95.5)	41(89.1)	
Missing	NA=5	NA=3	NA=2	
Disease type,n%				1
secondary	6(8.2)	2(8.0)	4(8.3)	
denovo	67(91.8)	23(92.0)	44(91.7)	
Cytogenetic risk-2022ELN				0.515
favorable	7(10.3)	1(4.5)	6(13.0)	
intermediate	55(80.9)	19(86.4)	36(78.3)	
poor	6(8.8)	2(9.1)	4(8.7)	
Missing	NA=5	NA=3	NA=2	
Common molecular mutation (%)				
KMT2A-PTD				0.112
positive	7(9.6)	0	7(14.6)	
negative	66(90.4)	25(100.0)	41(85.4)	
FLT3-ITD				0.161
Positive	19(26.0)	9(36.0)	10(20.8)	
Negative	54(74.0)	16(64.0)	38(79.2)	
ASXL1				1
Positive	8(11.0)	3(12.0)	5(10.4)	
Negative	65(89.0)	22(88.0)	43(89.6)	
CEBPA				0.112
Positive	7(9.6)	0	7(14.6)	
Negative	66(90.4)	25(100.0)	41(85.4)	
NRAS				0.836

Positive	5(6.8)	1(4.0)	4(8.3)	
Negative	68(93.2)	24(96.0)	44(91.7)	
DNMT3A				0.957
positive	10(13.7)	4(16.0)	6(12.5)	
negative	63(86.3)	21(84.0)	42(87.5)	
SRSF-2				0.076
Positive	5(7.0)	4(16.7)	1(2.1)	
Negative	66(93.0)	20(83.3)	46(97.9)	
Missing	NA=2	NA=1	NA=1	
BCORL1				0.221
positive	4(5.5)	3(12.0)	1(2.1)	
negative	69(94.5)	22(88.0)	47(97.9)	
IDH1				1
positive	4(5.5)	1(4.0)	3(6.3)	
negative	69(94.5)	24(96.0)	45(93.8)	
IDH2				0.006
Positive	5(6.8)	5(20.0)	0	
Negative	68(93.2)	20(80.0)	48(100.0)	
NPM1				0.328
Positive	8(11.0)	1(4.0)	7(14.6)	
Negative	65(89.0)	24(96.0)	41(85.4)	
TP53				0.067
positive	3(4.1)	3(12.0)	0	
negative	70(95.9)	22(88.0)	48(100.0)	

Abbreviations: CK, complex karyotype; CR1, first complete remission; HCT--CI, Hematopoietic Cell Transplant Comorbidity Index; HLA, human leucocyte antigen; HSCT, hematopoietic stem cell transplant; MRD, minimal residual disease.

Supplementary Table 3. Univariate analysis of OS, LFS, CIR, NRM, aGVHD, cGVHD and GRFS in the Haplo-HSCT cohort.

	CIR	P	NRM	P	LFS	P	OS	P	GRFS	P	Grade II–IV aGVHD	P	cGVHD	P
	**2years, % 95%CI**		**2years, % 95%CI**		**2years, % 95%CI**		**2years, % 95%CI**		**2years, % 95%CI**		**100days, % 95%CI**		**2years, % 95%CI**	
RUNX1 status		0.860		0.911		0.977		0.651		0.874		0.818		0.903
RUNX1-	6.9(0-14.6)		8.7(0.5-17)		84.4(74.4-95.8)		91.2(83.4-99.8)		65.2(52.7-80.7)		27.1(14.4-39.8)		44.6(28.9-60.2)	
RUNX1+	8.8(0-20.9)		8.8(0-20.9)		82.3(67.9-99.8)		85.8(72-100.0)		57.8(39.8-83.8)		20.0(4.0-36.0)		45.4(23.4-67.4)	
Age at HSCT		0.253		0.344		0.120		0.549		0.197		0.550		0.438
<38	5.7(0-13.4)		5.5(0-13.0)		88.9(79.1-99.8)		91.6(82.9-100.0)		72.5(59.2-88.6)		28.9(14.3-43.6)		40.9(23.5-58.3)	
≥38	10.0(0-21.1)		12.0(0.7-23.3)		78.0(64.6-94.1)		87.6(76.8-99.8)		52.9(37.6-74.4)		20.0(6.5-33.5)		51.2(31.5-70.9)	
Sex		0.296		0.319		0.136		0.237		0.08		0.720		0.913
female	4.0(0-11.8)		4.2(0-12.4)		91.8(81.6-100)		95.7(87.7-100.0)		79.9(65.5-97.4)		26.9(9.5-44.4)		45.5(22.7-68.3)	
Male	9.5(0.5-18.6)		11.1(1.8-20.4)		79.4(68.2-92.5)		86.3(76.7-97.2)		53.9(40.5-71.6)		23.4(11.1-35.7)		46.0(29.9-62.0)	
Transplantation		0.180		0.002		<0.001		<0.001		0.019		0.799		0.919
CR1	4.5(0-10.6)		2.2(0-6.4)		93.4(86.4-100.0)		97.8(93.6-100.0)		70.4(58.3-84.9)		23.5(11.8-35.3)		46.6(31.2-62.0)	
Non-CR1	15.1(0-31.5)		23.8(5-42.6)		61.1(43.2-86.5)		70.1(52.5-93.6)		45.8(28.3-74.2)		27.3(8.2-46.4)		42.6(18.3-66.9)	
Donor-recipient blood type match		0.815		0.171		0.390		0.317		0.493		0.693		0.567
Matched	7.3(0-15.4)		4.7(0-11.2)		88.0(78.6-98.5)		92.4(84.5-100.0)		68.2(55.2-84.3)		27.3(13.9-40.6)		42.4(25.2-59.5)	
Mismatched	7.9(0-18.7)		14.4(1.1-27.8)		77.7(63.3-95.3)		85.1(72.7-99.7)		57.3(41.6-79.1)		20.7(5.7-35.7)		49.3(29.3-69.3)	

	CIR	P	NRM	P	LFS	P	OS	P	GRFS	P	Grade II–IV aGVHD	P	cGVHD	P
	**2years, % 95%CI**		**2years, % 95%CI**		**2years, % 95%CI**		**2years, % 95%CI**		**2years, % 95%CI**		**2years, % 95%CI**		**2years, % 95%CI**	
Time from diagnosis to HSCT		0.898		0.368		0.448		0.620		0.701		0.844		0.747
<211	8.5(0-17.9)		5.7(0-72.3)		85.8(74.9-98.2)		91.2(82.1-100.0)		65.3(51.2-83.4)		25.0(10.6-39.4)		47.3(29.7-64.8)	
≥211	6.7(0-16.1)		0.2(0-11.0)		81.7(69.4-96.3)		88.4(78.3-99.8)		61.2(46.5-80.7)		24.3(10.3-38.4)		42.6(23.7-61.6)	
Sex match		0.782		0.938		0.767		0.966		0.192		0.891		0.256
Any other	7.2(0.3-14.1)		8.5(1.3-15.8)		84.3(75.3-94.3)		89.5(81.8-97.8)		66.6(55.2-80.2)		24.6(13.7-35.5)		42.1(28.3-55.8)	
Male recipient–female donor	9.1(0-26.9)		9.1(0-27.0)		81.8(61.9-100.0)		90.9(75.4-100.0)		47.6(25.7-88.2)		25.0(0-50.7)		66.0(29.8-100.0)	
HCT-CI		0.392		0.12		0.528		0.180		0.661		0.304		0.498
0-2	8.6(1.3-15.9)		6.6(0.3-13.0)		84.8(76.1-94.5)		91.3(84.3-98.9)		64.8(53.6-78.3)		26.6(15.6-37.5)		46.7(32.9-60.6)	
>3	0		22.2(0-51.2)		77.8(54.9-100.0)		77.8(54.9-100.0)		55.6(31-99.7)		11.1(0-32.9)		36.1(0-73.2)	
Pre-MRD		0.357		0.368		0.130		0.430		0.123		0.841		0.906
Negative	5.9(0-12.4)		7.2(0.3-14.1)		86.9(78.3-96.5)		90.8(83.3-98.8)		66.7(55.0-80.8)		23.7(12.8-34.7)		45.7(31.6-59.9)	
Positive	14.3(0-33.4)		14.3(0-33.4)		71.4(51.3-99.5)		85.1(68.0-100.0)		49.0(28.4-84.5)		28.6(3.9-53.2)		42.7(11.3-74.1)	
CK		0.573		0.477		0.328		0.467		0.358		0.214		0.820
NO	7.2(0.3-14.1)		8.6(1.3-15.8)		84.3(75.3-94.3)		91.2(84.2-98.9)		65.3(53.9-79.1)		24.2(13.4-35.0)		45.4(30.9-59.9)	

YES	0		16.7(0-49.3)		83.3(58.3-100.0)		83.3(58.3-100.0)		50.0(22.5-100.0)		50.0(3.2-96.8)		50.0(3.2-96.8)	

	**CIR**	**P**	**NRM**	**P**	**LFS**	**P**	**OS**	**P**	**GRFS**	**P**	**Grade II–IV aGVHD**	**P**	**cGVHD**	**P**
	**2years, % 95%CI**		**2years, % 95%CI**		**2years, % 95%CI**		**2years, % 95%CI**		**2years, % 95%CI**		**2years, % 95%CI**		**2years, % 95%CI**	
Disease type		0.487		0.444		0.297		0.410		0.322		0.139		0.762
denovo	8.2(1.2-15.2)		9.4(2.2-16.6)		82.4(73.4-92.5)		88.7(81.1-97.0)		61.7(50.6-75.2)		26.9(16.2-37.6)		41.1(28.5-53.8)	
secondary	0		0		100.0		100.0		80.0(51.6-100.0)		0		37.5(0-85.1)	
Cytogenetic risk		0.674		0.572		0.548		0.568		0.187		0.705		0.551
favorable	0		16.7(0-49.3)		83.3(58.3-100.0)		0		71.4(44.7-100.0)		14.3(0-42.3)		50.0(3.2-96.8)	
intermediate	7.9(0.3-15.5)		7.6(0.4-14.9)		84.5(75.1-95.1)		92.2(85.1-99.9)		66.9(55.0-81.3)		27.3(15.4-39.2)		40.3(26.1-54.6)	
poor	0		16.7(0-49.3)		83.3(58.3-100.0)		0		33.3(10.8-100.0)		33.3(0-76.0)		50.0(26.4-73.6)	
NRAS		0.270		0.341		0.135		0.434		0.303		0.180		0.635
negative	6.2(0.3-12.2)		7.8(1.2-14.3)		86.0(77.9-95.0)		90.3(83.2-98.0)		65.9(55.2-78.7)		26.5(15.9-37.0)		40.7(28.1-53.3)	
positive	26.7(0-80.5)		20.0(0-59.2)		53.3(21.4-100.0)		80.0(51.6-100.0)		53.3(21.4-100.0)		0		60.0(5.6-100.0)	
ASXL1		0.703		0.623		0.514		0.750		0.303		0.954		0.180
negative	8.5(1.3-15.7)		8.1(1.2-15)		83.4(74.5-93.4)		89.9(82.4-97.9)		67.6(56.8-80.5)		24.6(14-35.2)		42.5(28.9-56.1)	
positive	0		12.5(0-37.0)		87.5(67.3-100.0)		87.5(67.3-100.0)		45.0(19.8-100.0)		25.0(0-57.5)		70.8(27.6-100.0)	
DNMT3A		0.736		0.867		0.701		0.928		0.771		0.055		0.45
negative	7.1(0.3-13.9)		8.3(1.3-15.4)		84.6(75.7-94.4)		89.8(82.3-97.9)		62.6(51.2-76.5)		20.6(10.6-30.7)		41.7(28.6-54.8)	

positive	10.0(0-29.6)		10.0(0-29.7)		80.0(58.7-100.0)		88.9(70.6-100.0)		68.6(44.5-100.0)		50.0(16.6-83.4)		88.9(15.8-100.0)	

	**CIR**	**P**	**NRM**	**P**	**LFS**	**P**	**OS**	**P**	**GRFS**	**P**	**Grade II–IV aGVHD**	**P**	**cGVHD**	**P**
	**2years, % 95%CI**		**2years, % 95%CI**		**2years, % 95%CI**		**2years, % 95%CI**		**2years, % 95%CI**		**2years, % 95%CI**		**2years, % 95%CI**	
SRSF-2		0.531		0.346		0.768		0.426		0.816		0.772		0.364
negative	6.4(0.3-12.5)		8.0(1.2-14.7)		85.6(77.4-94.8)		90.1(82.8-98.0)		66.6(55.9-79.5)		25.8(15.1-36.4)		41.5(28.8-54.3)	
positive	0		20.0(0-59.2)		80.0(51.6-100.0)		80.0(51.5-100.0)		53.3(21.4-100.0)		20.0(0-59.2)		26.7(0-80.5)	
FLT3-ITD		0.659		0.722		0.521		0.850		0.043		0.971		0.866
negative	6.5(0-13.6)		7.9(0.4-15.4)		85.7(76.3-96.2)		89.9(81.9-98.7)		69.8(58.0-84.0)		24.1(12.5-35.6)		46.2(31.2-61.1)	
positive	10.5(0-24.7)		10.5(0-24.8)		79.0(62.6-99.6)		88.5(74.8-100.0)		45.0(26.8-75.6)		26.3(5.9-46.7)		42.9(16.9-68.8)	
NPM1		0.319		0.08		0.578		0.129		0.428		0.112		0.431
negative	8.6(1.3-15.9)		6.5(0.3-12.8)		84.9(76.3-94.6)		91.5(84.6-99.0)		65.2(54.0-78.6)		21.5(11.5-31.6)		45.0(31.6-58.3)	
positive	0		25.0(0-57.3)		75.0(50.3-100.0)		75.0(50.3-100.0)		50.0(25.0-100.0)		50.0(12.0-88.0)		41.7(0-84.4)	

CIR: cumulative relapse incidence, NRM: non-relapse mortality, LFS: leukemia-free survival, OS: overall survival, GRFS: GvHD-free/relapse-free survival

Supplementary Table 4. Outcome among patients with de novo AML (n=79) and patients with intermediate-risk cytogenetics (n=55), and patients who received HLA-matched sibling HSCT(n=18) as of RUNX1 mutational status.

	CIR	NRM	LFS	OS	GRFS	Grade II–IV aGVHD	cGVHD
	**2years,** % 95%CI	**2years,** % 95%CI	**2years,** % 95%CI	**2years,** % 95%CI	**1year,** % 95%CI	**100days,** % 95%CI	**2years,** % 95%CI
**De novo AML(n=79)**							
**Overall Outcome**	9.4(2.7-16.2)	7.9(1.8-14.0)	82.7(74.5-91.7)	90.3(83.8-97.4)	65.3(55.3-77.2)	24.1(14.6-33.5)	48.2(36.2-60.3)
**RUNX1 status**							
RUNX1-	6.5(0-13.6)	8.2(0.4-15.9)	85.4(75.9-96.0)	87.0(74.2-100.0)	69.4(57.6-83.7)	17.9(3.4-32.3)	48.5(27.9-69.1)
RUNX1+	14.8(1.1-28.5)	7.7(0-18.1)	75.6(63.2-95.2)	91.8(84.4-99.8)	56.7(40.2-80.2)	27.5(15.1-39.8)	47.5(32.6-62.4)
P	0.252	0.859	0.451	0.671	0.459	0.574	0.815
**Intermediate-risk cytogenetics(n=55)**							
**Overall Outcome**	11.9(2.8-20.9)	5.8(0-12.2)	82.4（72.5-93.6）	94.0(84.6-100.0)	66.6（54.6-81.2）	25.5(13.8-37.1)	48.1(32.7-63.5)
**RUNX1 status**							
RUNX1-	12.9(0.8-24.9)	3.0(0-9.0)	84.1（72.2-98.0）	88.2(73.9-100.0)	68.9（54.6-87.1）	23.8(5.1-42.5)	36.9(13.9-59.8)
RUNX1+	9.5(0-22.4)	10.9(0-25.8)	79.6（63.4-99.9）	96.9(91.0-100.0)	62.3（43.2-90.0）	26.5(11.4-41.6)	53.0(33.7-72.3)
P	0.851	0.283	0.606	0.253	0.653	0.815	0.435
**HLA-matched sibling HSCT cohort(n=18)**							
**Overall Outcome**	17.4(0-36.0)	5.6(0-16.4)	77.0(59.6-99.6)	94.4(84.4-100.0)	66.7(48.1-92.4)	11.1(0-26.1)	69.3(45.0-93.5)
**RUNX1 status**							
RUNX1-	11.1(0-32.9)	0	88.9(70.6-100.0)	100.0	80.0(58.7-100.0)	10.0(0-29.6)	77.8(46.1-100.0)
RUNX1+	25.0(0-57.7)	12.5(0-37.0)	62.5(36.5-100.0)	87.5(67.3-100.0)	50.0(25.0-100.0)	12.5(0-37.0)	58.3(16.1-100.0)
P	0.406	0.264	0.157	0.264	0.151	0.715	0.460

CIR: cumulative relapse incidence, NRM: non-relapse mortality, LFS: leukemia-free survival, OS: overall survival, GRFS: GvHD-free/relapse-free survival.

Supplementary Table 5. Exploratory analysis of further genetically defined subgroups.

	CIR	NRM	LFS	OS	GRFS
	**2years, % 95%CI**	**2years, % 95%CI**	**2years, % 95%CI**	**2years, % 95%CI**	**2years, % 95%CI**
**RUNX1 status**					
RUNX1-ASXL1-	9.0(0.4-17.6)	6.4(0-13.5)	84.6(74.7-95.8)	100.0	68.8(56.7-83.3)
RUNX1-ASXL1+	0	12.5(0-37.0)	87.5(67.3-100.0)	83.8(70.3-99.8)	62.5(36.5-100.0)
RUNX1+ASXL1-	11.2(0-23.5)	11.2(0-23.5)	77.6(63.2-95.2)	87.5(67.3-100.0)	57.3(40.8-80.5)
RUNX1+ ASXL1+	20.0(0-59.2)	0	80.0(51.6-100.0)	93.6(86.8-100.0)	53.3(21.4-100.0)
P	0.767	0.769	0.806	0.567	0.863
**MDS-R**					
without MDS-R	9.6(0.5-18.7)	6.8(0-14.4)	83.6（73.1-95.5）	93.1(85.9-100.0)	66.6（54.1-82.1）
with MDS-R	9.4(0.5-18.2)	9.4(0.5-18.2)	81.3（70.4-93.9）	87.5(77.8-98.5）	61.3（48.0-78.4）
P	0.6	0.672	0.5	0.342	0.8

CIR: cumulative relapse incidence, NRM: non-relapse mortality, LFS: leukemia-free survival, OS: overall survival, GRFS: GvHD-free/relapse-free survival.
